# Early cataract surgery and affordable Sinskey hook goniotomy in Black and Afro-Latino glaucoma patients: a 6-month retrospective study

**DOI:** 10.3389/fopht.2024.1294651

**Published:** 2024-02-28

**Authors:** Daniel Laroche, Ayobami Adebayo, Aaron Brown, Chester Ng, Soheil Sakkari

**Affiliations:** ^1^ Department of Ophthalmology, Icahn School of Medicine at Mount Sinai and New York Eye and Ear Infirmary of Mount Sinai, New York, NY, United States; ^2^ Department of Ophthalmology, Advanced Eyecare of New York, New York, NY, United States; ^3^ Department of Ophthalmology, Albert Einstein College of Medicine, Bronx, NY, United States

**Keywords:** glaucoma, Sinskey hook, goniotomy, early cataract surgery, MIGS

## Abstract

**Aim:**

The purpose of this study was to determine the real-world efficacy of early phacoemulsification cataract surgery and goniotomy with a Sinskey hook in patients with glaucoma.

**Methods:**

This study was conducted at Advanced Eye Care of New York, a private practice located in Manhattan, NY. This was a single-center, retrospective study of predominantly Black and Afro-Latino patients with glaucoma. These patients underwent early phacoemulsification cataract surgery and goniotomy using an affordable and reusable straight Sinskey hook (Ambler 200-μm tip). Patients who underwent the aforementioned procedure with 6 months of follow-up were included in this study. Investigated parameters were intraocular pressure, number of medications, mean deviation on visual field test, visual acuity, adverse events, and pre/postoperative spherical refractive error.

**Results:**

Among all 38 eyes that were enrolled in the study and underwent surgery (goniotomy using a Sinskey hook with phacoemulsification), mean intraocular pressure was reduced from 16.45 mmHg at baseline to 13.24 mmHg at month 6, a 19.5% reduction. The mean number of topical intraocular pressure-lowering medications used was reduced from 1.81 at baseline to 0.52 at month 6, a 71% reduction in topical medications.

**Conclusion:**

Combined early cataract surgery and goniotomy performed with a Sinskey hook is an affordable microinvasive surgery and an effective way to reduce intraocular pressure and the number of ocular hypertensive medications used in Black and Afro-Latino patients with primary open-angle glaucoma.

## Introduction

**Figure 1:**
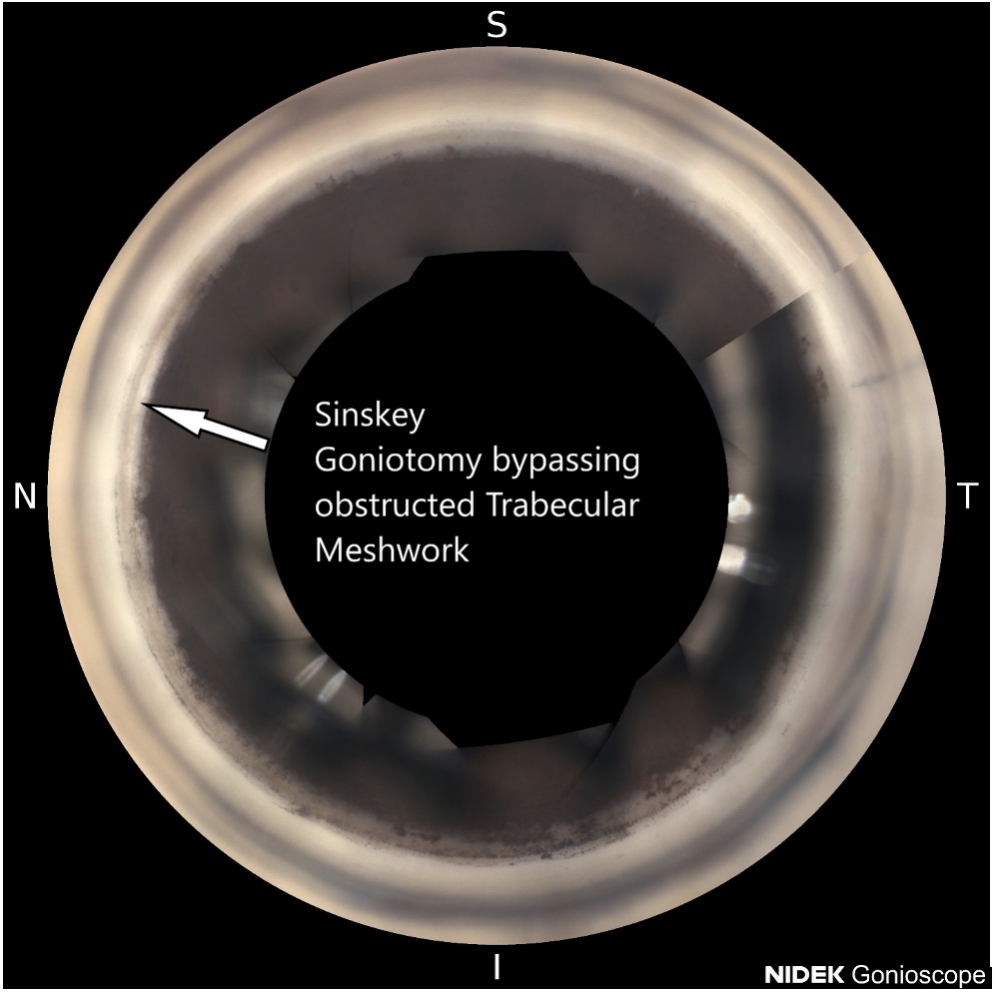
Sinskey goniotomy bypassing obstructed trabecular meshwork.

Glaucoma is a leading cause of preventable blindness and has a higher prevalence in the Black and Afro-Latino communities (1). The main risk factor for glaucoma is increased intraocular pressure (2). The enlargement of the lens is the most identifiable cause of glaucoma. Early cataract surgery/lens extraction combined with trabecular bypass should be considered as an initial approach for most people with glaucoma over the age of 50 (3). Black and Afro-Latino communities globally have less ready access to cataract surgery and glaucoma surgery than white communities (4). The thickness of the lens increases with age (3). When the size of the lens increases, during accommodation, the lens size increases further, thereby contacting the posterior bowing of the iris, leading to rubbing of the iris and pigment liberation (3). This excessive pigment liberation obstructs the trabecular meshwork, leading to increased stiffness and resistance (**Figure 1**).

The increase in lens size can also lead to pupillary block and narrow-angle or angle-closure glaucoma. These are the most common identifiable factors that lead to increased trabecular meshwork stiffness, leading to decreased outflow of aqueous humor. In the Baltimore Eye Survey, patients with untreated glaucoma had an average intraocular pressure of 18 mmHg, whereas those without glaucoma had an average pressure of 15 mmHg (5). Glaucoma medications are useful in reducing the rate of visual field loss. However, this does not fully halt the progression of glaucoma. With increased missed doses of medication, there is increased progression of visual field loss (6). In addition, there are tremendous financial inequalities in Black and Afro-Latino communities, making it difficult for this population to adhere to the medication regimens prescribed by their physicians (7). There is also a shortage of healthcare facilities and skilled eye surgeons in these communities globally (7). If cataract surgery is performed earlier in a patient’s life, this can often reduce intraocular pressure and prevent glaucoma (8).

Microinvasive glaucoma surgery has recently increased in popularity. This approach offers patients safer surgery than trabeculectomy to reduce intraocular pressure (IOP). It offers faster visual recovery, a reduced number of secondary interventions such as suture lysis, and fewer complications. Recent trials have suggested that lens extraction through cataract surgery is even more effective in reducing intraocular pressure when compared with peripheral iridotomy (9). Cataract surgery and the Hydrus stent have been demonstrated to reduce IOP and preserve the visual field (8).

Many minimally invasive glaucoma surgery (MIGS) devices are expensive, such as the Hydrus, iStent, and Omni. In a recent study, the Kahook Dual Blade was found to be the most cost-effective MIGS device (10). Tanito (9) has also developed a trabecular hook to perform goniotomy that has been shown to be similar to the Kahook Dual Blade (11).

**Figure 2:**
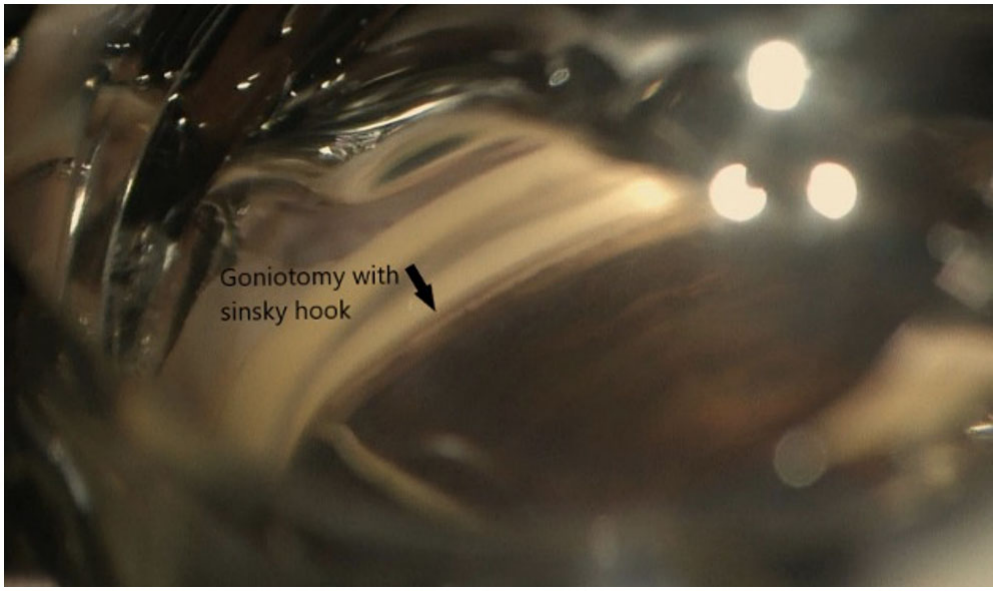
Goniotomy with Sinskey hook.

**Figure 3:**
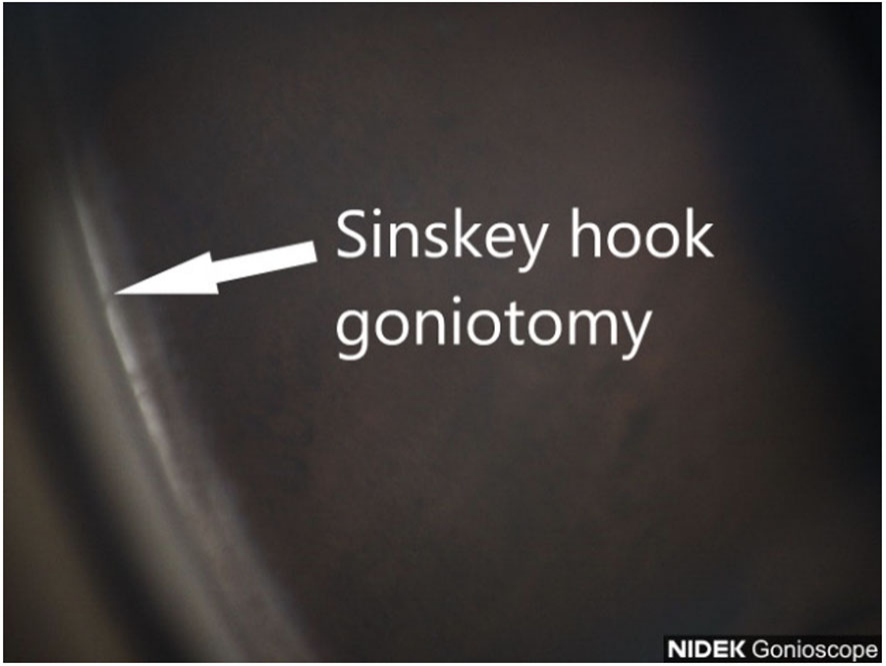
Sinskey hook goniotomy.

We previously reported the use of a straight 23-gauge cystotome to perform affordable goniotomy (10). In this article, we report our experience with the use of another inexpensive device that can be used to perform MIGS at the time of cataract surgery, the Sinskey hook (**Figures 2**, **3**). Its advantage is its 200-μm tip, which is compatible with the size of the trabecular meshwork. The tip is also smooth, meaning that once it is inserted into Schlemm’s canal, it does not damage the back wall and can remove the stiff inner wall of Schlemm’s canal and the overlying stiff juxtacanalicular meshwork. This reduces the risk of bleeding due to injury to the back wall of Schlemm’s canal and the nearby ciliary body. We examined the outcomes of early cataract surgery and goniotomy performed with a Sinskey hook. The safety and efficacy after 6 months were evaluated.

## Methods

This study was a retrospective report on Black and Afro-Latino patients (from the local community that makes up the practice in Harlem, New York City) with open-angle glaucoma who underwent phacoemulsification cataract surgery and goniotomy using a Sinskey hook with an Ambler 200-μm tip Ambler Surgical, Exton, PA, USA. This study involved human subjects and was conducted in accordance with the ethical standards of the Icahn School of Medicine Institutional Review Board. A waiver of informed consent was approved by the New York Eye and Ear Infirmary of Mount Sinai Institutional Review Board (IRB) due to the retrospective nature of the study. Retrospective information was collected from a single practice in New York, USA.

Data were collected preoperatively, postoperatively, after 1 day, after 1 month, and after 6 months during each patient’s clinical course. The goal for all patients included was to achieve a significant reduction in intraocular pressure from the baseline. However, each patient’s situation and target intraocular pressure were different, just as their baseline pressure measurements were. During assessments, the need to add or remove medication was considered and this was performed as needed.

The straight Sinskey hook is a reusable ophthalmic instrument that is commonly used in cataract removal procedures. This is the tool that was used to remove the trabecular meshwork and the inner wall of Schlemm’s canal.

The study’s primary outcome measure was observation of a reduction in intraocular pressure compared with the preoperative baseline. Six outcome measures were evaluated in total: reduction in intraocular pressure, reduction in the amount of medication used, visual field, visual acuity, adverse events, and pre/postoperative spherical refractive error.

## Procedure

**Figure 4:**
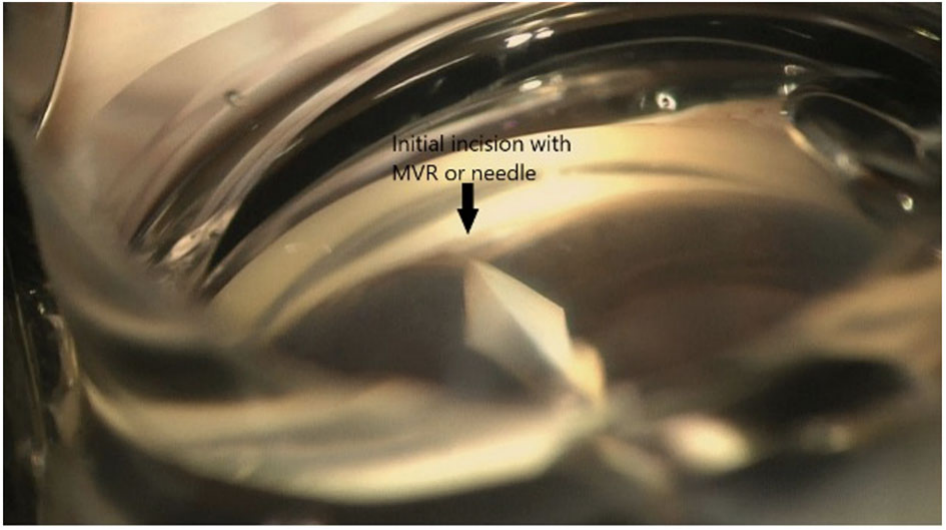
Initial incision with MVR or needle.

Patients were administered preoperative prednisolone acetate 1% (Allergan, Dublin, Ireland) QID and ofloxacin (Rising, Saddle Brook, NJ, USA) QID and Ketorolac 0.4% (Allergan, Dublin Ireland) TID starting 3 days prior to surgery. After the eye was prepared with betadine and draped, topical anesthesia was applied, and clear corneal phacoemulsification was performed with implantation of an intraocular lens. EndoCoat (Abbott, Chicago, IL, USA) was placed in the eye to deepen the angle. The patient’s head was tilted away from the surgeon at approximately 45°, and the microscope was tilted toward the surgeon at approximately 45°. A direct gonio lens (Katena, Troy Hills, NJ, USA) was placed on the eye, and the microscope was focused down to obtain a direct view of the nasal angle structures. An Microvitreoretinal (MVR) blade was used to pass through the layers of the trabecular meshwork and enter Schlemm’s canal (**Figure 4**). The Sinskey hook was then used to enter the canal, and a pass to the left by 2–3 clock-hours was performed to unroof Schlemm’s canal. Subsequently, the Sinskey hook was passed to the right to unroof another 2–3 clock-hours of the canal (**Figure 2**). The device was then withdrawn, and the ophthalmic viscosurgical device was removed and replaced with a balanced salt solution. Intracameral injection of diluted Vigamox (Alcon, Geneva, Switzerland) with 1 cc of balanced saline solution 50/50 was administered *via* paracentesis at the end of the procedure. In all patients, we observed that the angle was open nasally after cataract surgery. Postoperatively, patients were instructed to sleep in a sitting position for four nights to allow any heme reflux to settle, clear, and avoid obstructing the visual axis. Patient were instructed to continue Ofloxacin for 7 days, continue Prednisolone acetate QID for 1 week, then TID for 1 week then BID for 1 week then qday for 1 week then discontinue. The patient was instructed to use Ketorolac TID for 4 weeks then discontinue. The patient was instructed to discontinue glaucoma medications in the operated eye.

## Results

A total of 38 eyes were enrolled in the study, with 38 eyes undergoing follow-up at 6 months. Data for the corresponding patients on decrease in intraocular pressure and number of medications were included in the analysis (**Table 1**).

**Table 1:** Number of glaucoma medications taken and intraocular pressure at baseline and follow-up times for all patients (38) who underwent Sinskey hook goniotomy and phacoemulsification cataract surgery.

Timepoint	Intraocular pressure (IOP), mmHg		Ocular hypertensive medications		BCVA	Visual field test
	Mean ± SD	Median (IQR)	Mean ± SD	Median (IQR)	LogMAR (mean ± SD)	Mean deviation ± SD
Baseline	16.45 ± 4.8	16.5 (5–28)	1.81 ± 1.5	1 (0–7)	0.15 ± 0.34	−7.64 ± 8.1
Postoperative day 1	13.82 ± 6.9	12 (5–45)	0	0 (0)	-	
1 month	16.63 ± 5.9	15 (7–41)	0.26 ± 1.0	0 (0–7)	0.12 ± 0.25	-
3 months	14.34 ± 5.1	14 (7–43)	0.40 ± 1.0	0 (1–4)	0.14 ± 0.26	-
6 months	13.24 ± 3.04	13 (7–22)	0.52 ± 1.2	0 (0–4)	0.14 ± 0.28	-

BCVA, best corrected visual acuity; IQR, interquartile range; SD, standard deviation.

**Figure 5:**
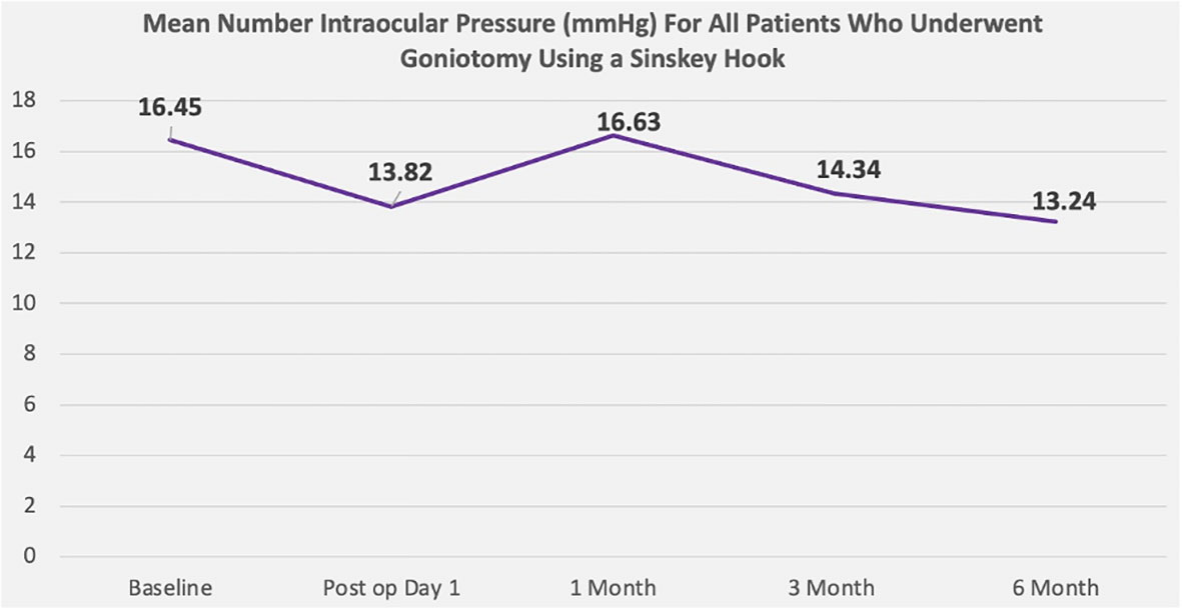
Mean intraocular pressure (over the course of 6 months) for patients who underwent goniotomy using a Sinskey hook.

**Figure 6:**
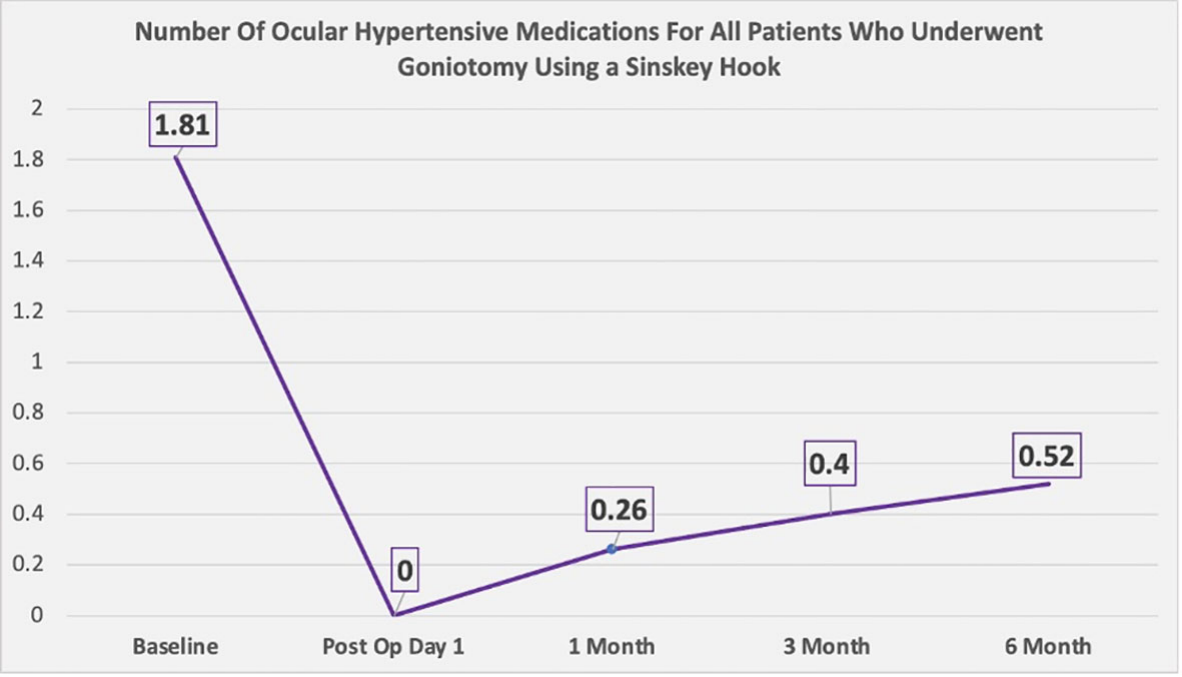
Number of ocular hypertensive medications used (over the course of 6 months) by patients who underwent goniotomy using a Sinskey hook.

**Figure 7:**
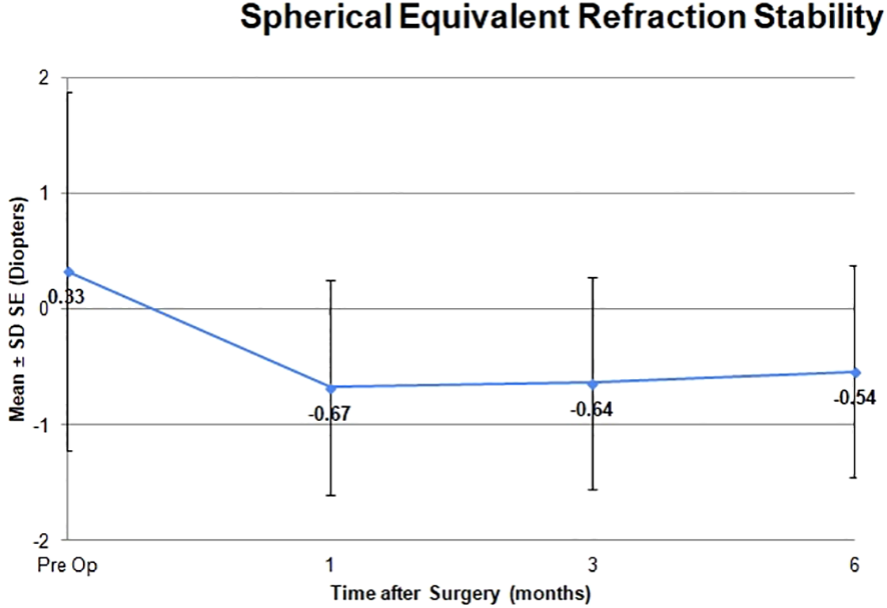
Spherical equivalent refraction stability.

The mean age among patients was 65 ± 9 years, 42% were men and 58% were women (**Table 2**). Twenty-one patients had primary open-angle glaucoma, seven had ocular hypertension, eight had angle-closure glaucoma, and two had pigmentary glaucoma (**Table 3**). Goniotomy using a Sinskey hook and phacoemulsification cataract surgery significantly decreased intraocular pressure in these patients. The mean ± standard deviation intraocular pressure at baseline for all eyes was 16.45 ± 4.8 mmHg. After 6 months, the mean ± standard deviation intraocular pressure was 13.24 ± 3.0 mmHg (**Figure 5**). A statistically significant reduction in intraocular pressure was observed on postoperative day 1, and the reduction remained significant at every postoperative timepoint through 6 months of follow-up, except for the first month. From baseline to 6 months, there was a reduction in intraocular pressure by >19.5% in all eyes. There was also a decrease in the number of medications used over the 6-month follow-up period (**Figure 6**). At baseline, the mean ± standard deviation number of ocular hypertensive medications used was 1.81 ± 1.5. After 6 months, the mean ± standard deviation number of ocular hypertensive medications used was 0.52 ± 1.2. Vision in patients was stable, with LogMAR best corrected visual acuity (BCVA) ranging from 0.15 ± 0.34 to 0.14 ± 0.28 (**Table 1** and **Figure 7**). At 6 months, medication was no longer used in 78% (30/38) of the eyes included in this study.

**Table 2:** Characteristics of patients who underwent goniotomy using a Sinskey hook combined with phacoemulsification cataract surgery.

Variable	Category	Statistics
**Age (years)**	Mean ± SD	65 ± 9
**Gender, *n* (%)**	Male	16/38 (42%)
	Female	22/38 (58%)
**Eye, *n* (%)**	Right	18/38 (47%)
	Left	20/38 (53%)
**Baseline IOP (mmHg)**	Mean ± SD	16.45 ± 4.8
**Ocular hypertensive medications**	Mean ± SD	1.81 ± 1.5
**Number of ocular hypertensive medications used, (%)**	0	16%
	1	43%
	2	4%
	3	23%
	≥4	15%
**Visual acuity (LogMar)**	Mean ± SD	0.15 ± 0.34
**MD on VFT**	Mean ± SD	(−7.64) ± 8.1
**VFI on VFT**	Mean ± SD	79.32 ± 29.04

MD, mean deviation; SD, standard deviation; VFI, visual field index; VFT, visual function test; IOP, intraocular pressure.

**Table 3:** Glaucoma diagnoses for all eyes included.

Diagnosis	No. of eyes
Primary open-angle glaucoma	21
Ocular hypertension	7
Angle-closure glaucoma	8
Pigmentary glaucoma	2

**Figure 8:**
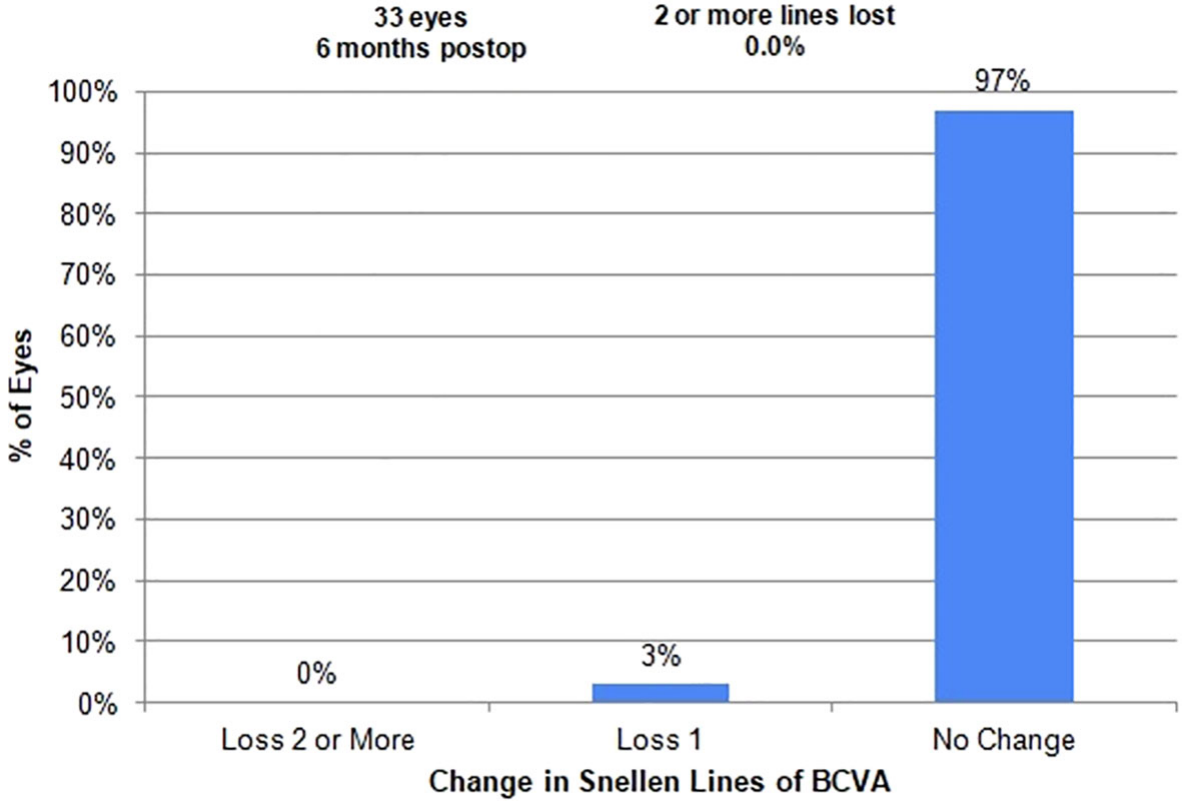
Snellen visual acuity.

The adverse effects observed were minor and posed no threat to the sight of the patients. There were two eyes in which transient hyphema was noted, but this can occur during an excisional goniotomy (**Table 4**). It is typical for the hyphema to clear within the first week after the excisional goniotomy, which was the case here. There were no cases of persistent hyphema or IOP spike that required an anterior chamber washout. There were no complications related to early cataract surgery. The mean spherical equivalent shifted from +0.33 to −0.54 and was stable at 6 months after surgery (**Table 5** and **Figure 8**). There was no change in visual acuity in 97% of patients (**Table 1**). One patient developed an epiretinal membrane with 20/40 vision.

**Table 4:** Adverse effects of goniotomy using a Sinskey hook combined with phacoemulsification cataract surgery.

Adverse effect	Number	Percentage
**Hyphema**	2	2.5

**Table 5:** Mean spherical equivalents for all eyes included.

Spherical equivalent (SE) category	Number
Mean preoperative SE (mmHg)	0.33 ± 2.22
Mean postoperative SE (mmHg)	−0.54 ± 0.81

## Discussion

This study demonstrates the effectiveness of early cataract surgery and affordable goniotomy using a Sinskey hook in improving vision, reducing intraocular pressure, and decreasing the number of ocular hypertensive medications needed 6 months later. Most of the patients in this study had moderately advanced glaucoma on presentation (mean deviation on Humphrey visual field, −7.64 ± 8.1) (**Table 2**).

Uncomplicated early cataract surgery and excisional goniotomy should be considered earlier and as an initial treatment for patients with age-related enlarged lens-induced glaucoma. Although pharmacologic agents used to reduce intraocular pressure have been demonstrated to reduce the rate of visual field loss, these agents do not fully halt the progression of glaucoma. High-risk sociodemographic groups such as Black and Afro-Latino patients also face many financial barriers that reduce their capacity to adhere to medication regimens. Many of these patients also have difficulty with compliance.

The Sinskey hook is a very cost-effective device for excisional goniotomy in comparison with other minimally invasive glaucoma surgery devices. A study performed by Chen et al. was able to highlight the cost-effectiveness of the Kahook Dual Blade. In this study, the authors compared the iStent Inject, the Kahook Dual Blade, the Trabectome, and the Hydrus Microstent. Each device was compared based on cost-effectiveness in terms of cost per mmHg of intraocular pressure reduction (12). Although each device achieved similar outcomes when used during surgery, the cost of using each device varied significantly. Chen et al. concluded that the Kahook Dual Blade was the most cost-effective in terms of cost per mmHg of intraocular pressure reduction. Following the Kahook Dual Blade were the Hydrus Microstent and then the Trabectome. Finally, the iStent Inject was found to be the most expensive of the devices studied for reducing intraocular pressure (12). We obtained similar results to those reported for the Kahook Dual Blade in terms of IOP reduction, at a lower cost again.

In a recent study on phacoemulsification combined with excisional goniotomy using the Kahook Dual Blade for cataract and open-angle glaucoma by Albuainain et al., similar intraocular pressure results were obtained. At months 24 and 36, the mean IOP was 13.9 (0.3) and 13.9 (0.5) mmHg, respectively, and the mean numbers of medications were 1.4 (0.2) and 2.0 (0.4), respectively (13).

The 5-year HORIZON trial revealed that cataract surgery with the Hydrus Microstent reduces the rate of visual field loss by 47% versus cataract surgery alone (14). Earlier results have demonstrated significant reductions in IOP, medication use, and secondary glaucoma surgeries. In the 5-year data, 73% of “mild” Microstent patients (those taking one glaucoma medication at baseline) remained medication-free at 5 years, compared with 48% in the arm of the study who underwent cataract surgery only. In addition, among the stent group, there was a more than 60% (2.8×) reduction in the likelihood of requiring subsequent invasive glaucoma surgery—a clinically meaningful and statistically significant difference. Use of the Hydrus achieved a 2.5% rate of subsequent invasive glaucoma surgery, compared with 6.4% for cataract surgery alone.

Globally, there is a wealth gap between Black people and White people. In the USA alone, the average net worth of a White family is $171,000.00 and that of a Black family is $17,000.00 (15). Poverty is even greater among the Black community outside the USA. This is because of the history of slavery and colonization that we have inherited and the structural institutions that remain. The Sinskey hook is often a standard part of cataract surgery sets, is often readily available, and can make goniotomy more available in resource-poor areas globally.

Since cataract surgery in skilled hands has become much safer over the last 25 years, early cataract surgery combined with Sinskey hook goniotomy MIGS can be considered as an earlier treatment option in patients with glaucoma. Although there were no complications related to early cataract surgery, further research with a larger group is required. The age of our patients was 65 years, which is 8 years younger than the average age for cataract surgery in the USA. This approach can also be considered as a safer first-line therapy in patients with mild-to-moderate glaucoma. However, insurance companies usually deny health care providers the chance to perform these surgeries before deterioration in the patient’s vision occurs. Instead of pharmacologic agents, early cataract surgery should be considered as an initial option to reduce intraocular pressure and the risk of blindness in patients with glaucoma, particularly for Black and Afro-Latino patients. A more affordable option will also allow these surgeries to be performed in places such as sub-Saharan Africa, where glaucoma is extremely prevalent but most patients cannot afford extremely expensive procedures to preserve their vision. Along with high costs, there is also a lack of surgical equipment for MIGS. Sinskey goniotomy can also be performed with manual small-incision cataract surgery (MSICS), which has recently gained popularity in Sub-Saharan Africa and other underserved areas (16). A study by Okuda recently found that the longer glaucoma patients are on medications, the lower their success rate with goniotomy. Among patients who have been on medications for longer than 4.5 years, there is a higher failure rate compared with those who have been on medications for less than 4.5 years (17). This supports our approach of earlier surgery. A recent study by Mansberger revealed that goniotomy performed at the time of cataract surgery reduces intraocular pressure spikes by 70% (18). Our study supports this report, as we observed only a small number of IOP spikes. Overall, we observed stable vision in this group of patients. One patient had decreased vision caused by an epiretinal membrane. The risks of cataract surgery must be discussed with patients, and potential postoperative complications must be avoided and managed properly to ensure an excellent outcome.

## Conclusion

This study demonstrates that early cataract surgery and goniotomy using a Sinskey hook is an effective and more affordable way to reduce intraocular pressure in Black and Afro-Latino patients with glaucoma. Other MIGS methods are costly and should not be seen as the only options for performing excisional goniotomy. This approach is also effective in reducing the amount of ocular hypertensive medication required by patients with glaucoma. Longer-term follow-up and larger sample sizes, including Black and Afro-Latino patients, are required to better evaluate the effectiveness of this method. Furthermore, more studies should be completed to add to the data suggesting that excisional goniotomy and cataract extraction should be considered as a first line of treatment in reducing intraocular pressure and the number of medications needed.

## Limitations

Aspects of the study that can be considered limitations are the fact that it was non-randomized, with a small sample size, and lacked a control group and longer-term follow-up. A comparative study is recommended with follow-up of 1 year and could further support these conclusions.

## Data availability statement

The raw data supporting the conclusions of this article will be made available by the authors, without undue reservation.

## Ethics statement

The studies involving humans were approved by Icahn School of Medicine Institutional Review Board. The studies were conducted in accordance with the local legislation and institutional requirements. The participants provided their written informed consent to participate in this study.

## Author contributions

DL: Conceptualization, Methodology, Writing – review & editing. AA: Formal analysis, Writing – original draft, Writing – review & editing. AB: Software, Writing – review & editing. CN: Data curation, Writing – review & editing. SS: Software, Writing – review & editing.
